# Phylogeny and taxonomy of *Laetiporus* (Basidiomycota, Polyporales) with descriptions of two new species from western China

**DOI:** 10.3897/mycokeys.37.26016

**Published:** 2018-07-31

**Authors:** Jie Song, Yi-Fei Sun, Xing Ji, Yu-Cheng Dai, Bao-Kai Cui

**Affiliations:** 1 Institute of Microbiology, Beijing Forestry University, Beijing 100083, China; 2 Beijing advanced innovation centre for tree breeding by molecular design, Beijing Forestry University, Beijing 100083, China; 3 Key Laboratory of State Forestry Administration on Tropical Forestry Research, Research Institute of Tropical Forestry, Chinese Academy of Forestry, Guangzhou 510520, China

**Keywords:** Brown-rot fungi, multi-gene phylogeny, Fomitopsidaceae, taxonomy, wood-decaying fungi

## Abstract

*Laetiporus* is a cosmopolitan genus of brown rot fungi. In this study, *L.medogensis* and *L.xinjiangensis* are described as new species from western China, based on morphological and molecular evidence. *L.medogensis* has only been found on gymnosperms so far and is distinguished by pinkish-buff to clay-buff pileal surface and buff-yellow pore surface, azonate to faintly zonate pileus and ellipsoid to ovoid basidiospores (5–6.2 × 4.2–5.2 μm). *L.xinjiangensis* is found on angiosperms and is characterised by pale-buff to clay-pink pileal surface, cream to light yellow pore surface, azonate to faintly zonate pileus, large pores (2–3 per mm) and small basidiospores (4.5–5 × 3–4.2 μm). The phylogeny of *Laetiporus* is reconstructed with multi-gene sequences including the internal transcribed spacer regions (ITS), the large subunit (nrLSU) and small subunit (nrSSU) of the nuclear ribosomal RNA gene, the small subunit of the mitochondrial rRNA gene (mtSSU), the translation elongation factor 1-α gene (EF-1α) and the second subunit of RNA polymerase II (RPB2). The results show that *L.medogensis* and *L.xinjiangensis* formed two distinct lineages belonging to *Laetiporus*. Illustrated descriptions of the two new species are presented. An identification key to species of *L.sulphureus* complex is provided.

## Introduction

*Laetiporus* Murrill (Fomitopsidaceae, Polyporales) is a cosmopolitan genus, causing brown rot on living hardwoods and conifers (Murrill 1904). Some species of the genus are known as forest pathogens and some are edible with medicinal functions (Dai et al. 2007, 2009). According to previous studies, 15 species have been accepted in the genus worldwide and 11 species have been confirmed in the *L.sulphureus* complex by phylogenetic analyses, of which six have been reported from China: *L.ailaoshanensis* B.K. Cui & J. Song, *L.cremeiporus* Y. Ota & T. Hatt., *L.montanus* Černý ex Tomšovský & Jankovský, *L.sulphureus* (Bull.) Murrill, *L.versisporus* (Lloyd) Imazeki and *L.zonatus* B.K. Cui & J. Song (Tomšovský and Jankovský 2008, Ota et al. 2009, Banik et al. 2012, Song et al. 2014, Song and Cui 2017). The species in the *L.sulphureus* complex are characterised by annual basidiocarps, soft and fleshy context and a dimitic hyphal system composed of simple septate generative hyphae and binding hyphae (Burdsall and Banik 2001, Núñez and Ryvarden 2001, Ota et al. 2009).

A molecular phylogenetic study of *Laetiporus* in Japan identified three species, viz. *L.cremeiporus*, *L.montanus* and *L.versisporus* (Ota and Hattori 2008, Ota et al. 2009). Recently, systematic studies have been carried out to define the species and explore the historical biogeography of the genus *Laetiporus* in China. Song et al. (2014) described two new *Laetiporus* species from south-western China based on morphological and molecular evidence. Further comprehensive study of Song and Cui (2017) indicated that there are two additional undescribed *Laetiporus* species.

In the present study, the two new *Laetiporus* species from western China (Clade P and Clade Q) are described based on morphological and phylogenetic analyses.

## Materials and methods

### Morphological studies

Morphological studies followed Han et al. (2016). The studied specimens were deposited in the herbarium of the Institute of Microbiology, Beijing Forestry University (BJFC). Macro-morphological descriptions were based on field notes. Colour terms followed Petersen (1996). Microscopic measurements and drawings were made from slide preparations of dried specimens stained with Cotton Blue and Melzer’s reagent, following Han et al. (2016). Sections were studied at a magnification of 1000× using a Nikon Eclipse 80i microscope and phase contrast illumination. Drawings were made with the aid of a drawing tube. Spores were measured in tube sections. In presenting spore size variation, 5% of measurements were excluded from each end of the range and given in parentheses. The following abbreviations were used: KOH = 5% potassium hydroxide, CB = cotton blue, CB+ = cyanophilous, CB– = acyanophilous, IKI = Melzer’s reagent, IKI– = neither amyloid nor dextrinoid, L = mean spore length (arithmetic average), W = mean spore width (arithmetic average), Q = variation in the L/W ratios between specimens studied, n (a/b) = number of spores (a) measured from a given number of specimens (b).

### Molecular study and phylogenetic analysis

Genomic DNA was extracted from dried fruiting bodies using a cetyltrimethylammonium bromide rapid plant genome extraction kit (Aidlab Biotechnologies Co., Ltd., Beijing) according to the manufacturer’s instructions with some modifications (Han et al. 2016). Six genetic markers were used, including ITS, nrLSU, nrSSU, EF-1α, mtSSU and RPB2. The primer pairs ITS5/4, LR0R/LR7, MS1/MS2, NS1/NS4, 983F/1567R and 6F/7R were used to amplify ITS, nrLSU, mtSSU, nrSSU, EF-1α and RPB2, respectively (http://www.biology.duke.edu/fungi/mycolab/primers.htm). A 2 × EasyTaq PCR SuperMix (Transgen Biotech, Beijing) was used to amplify the genes. The PCR procedure for ITS, EF-1α, mtSSU and RPB2 was as follows: initial denaturation at 95 °C for 3 min, followed by 35 cycles at 94 °C for 40 s, 54 °C for 45 s and 72 °C for 1 min and a final extension of 72 °C for 10 min. The PCR procedure for nrLSU and nrSSU was as follows: initial denaturation at 94 °C for 1 min, followed by 35 cycles at 94 °C for 30 s, 50 °C for 1 min and 72 °C for 1.5 min and a final extension of 72 °C for 10 min. The PCR products were purified using the Bioteke DNA Purification Kit (Bioteke Corporation, Beijing) and sequenced at the Beijing Genomics Institute, China, with the same primers. The basic authenticity and reliability of newly generated sequences were established based on Nilsson et al. (2012). The newly generated sequences and additional sequences downloaded from GenBank (www.ncbi.nlm.nih.gov/genbank; Benson et al. 2017) are listed in Table 1. Sequences of ITS, nrLSU, nrSSU, EF-1α, mtSSU and RPB2 of species in *Laetiporus* and outgroups [*Antrodiaserialis* (Fr.) Donk and *Fomitopsispinicola* (Sw.) P. Karst.] were combined and aligned in MAFFT 7 (Katoh and Toh 2008; https://mafft.cbrc.jp/alignment/server/index.html) using the “G-INS-I” strategy and manually adjusted in BioEdit v7.2.6.1 (Hall 1999).

**Table 1.** A list of species, specimens and GenBank accession numbers of sequences used in this study.

Species	Collection no.	GenBank Accessions					
		ITS	nrLSU	nuSSU	mtSSU	EF-1α	RPB2
* Antrodia serialis *	Cui 10519	KP715307	KP715323	KR605911	KR606011	KP715337	KR610830
* Fomitopsis pinicola *	Cui 10405	KC844852	KC844857	KR605857	KR605961	KR610690	KR610781
* Laetiporus ailaoshanensis *	Dai 13567 (Paratype)	KX354470 ^a^	KX354498 ^a^	KX354535 ^a^	KX354577 ^a^	KX354623 ^a^	KX354665 ^a^
* L. ailaoshanensis *	Dai 13256 (Holotype)	KF951289 ^a^	KF951317 ^a^	KX354537 ^a^	KX354579 ^a^	KX354625 ^a^	KT894786 ^a^
* L. caribensis *	PR 6583	JN684766	-	-	-	-	-
* L. caribensis *	PR 914	JN684762	EU402526	-	EU402482	-	-
* L. caribensis *	PR 6521	JN684771	-	-	-	-	-
* L. cincinnatus *	Dai 12811	KF951291 ^a^	KF951304 ^a^	KX354516 ^a^	KX354558 ^a^	KX354605 ^a^	KT894788 ^a^
* L. cincinnatus *	DA 37	EU402557	EU402521	-	EU402485	AB472661	-
* L. cincinnatus *	JV 0709/168J	KF951290 ^a^	KF951305 ^a^	KX354517 ^a^	KX354559 ^a^	KX354606 ^a^	KX354651 ^a^
* L. conifericola *	JV 0709/81J	KF951292 ^a^	KF951327 ^a^	KX354531 ^a^	KX354573 ^a^		KX354683 ^a^
* L. conifericola *	CA 8	EU402575	EU402523	-	EU402487	AB472663	-
* L. conifericola *	JAM 1	EU402577	EU402524	-	EU402486	AB472664	-
* L. cremeiporus *	Dai 10107	KF951281 ^a^	KF951301 ^a^	KX354515 ^a^	KX354557 ^a^	KX354604 ^a^	KX354650 ^a^
* L. cremeiporus *	Cui 10991	KF951279 ^a^	KF951298 ^a^	-	KX354595 ^a^	KX354641 ^a^	KX354679 ^a^
* L. cremeiporus *	Cui 10586	KF951277 ^a^	KF951297 ^a^	KX354513 ^a^	KX354555 ^a^	KX354602 ^a^	KX354648 ^a^
* L. gilbertsonii *	JV 1109/31	KF951293 ^a^	KF951306 ^a^	KX354542 ^a^	KX354584 ^a^	KX354630 ^a^	KX354671 ^a^
* L. gilbertsonii *	TJV 2000/101	EU402553	EU402528	-	EU402493	AB472668	-
* L. gilbertsonii *	CA 13	EU402549	EU402527	-	EU402496	AB472666	-
* L. huroniensis *	HMC 3	EU402571	EU402540	-	-	-	-
* L. huroniensis *	MI 14	EU402573	EU402539	-	EU402489	AB472672	-
* L. medogensis *	Cui 12219 (Paratype)	KX354472 ^a^	KX354500 ^a^	KX354538 ^a^	KX354580 ^a^	KX354626 ^a^	KX354667 ^a^
* L. medogensis *	Cui 12240 (Holotype)	KX354473 ^a^	KX354501 ^a^	KX354539 ^a^	KX354581 ^a^	KX354627 ^a^	KX354668 ^a^
* L. medogensis *	Cui 12390 (Paratype)	KX354474 ^a^	KX354502 ^a^	KX354540 ^a^	KX354582 ^a^	KX354628 ^a^	KX354669 ^a^
* L. montanus *	Dai 15888	KX354466 ^a^	KX354494 ^a^	KX354530 ^a^	KX354572 ^a^	KX354619 ^a^	KX354662 ^a^
* L. montanus *	Cui 10011	KF951274 ^a^	KF951315 ^a^	KX354528 ^a^	KX354570 ^a^	KX354617 ^a^	KT894790 ^a^
* L. montanus *	Cui 10015	KF951273 ^a^	KF951311 ^a^	KX354529 ^a^	KX354571 ^a^	KX354618 ^a^	KT894791 ^a^
*L.* sp. 1	EUC 1	EU402545	EU402541	-	-	-	-
*L.* sp. 1	KOA 1	EU402546	EU402542	-	-	-	-
*L.* sp. 2	RV4A	EU840662	-	-	-	-	-
*L.* sp. 2	RV5A	EU840663	-	-	-	-	-
*L.* sp. 3	Munez 207	JN684764	-	-	-	-	-
*L.* sp. 4	Robledo 1122	JN684765	-	-	-	-	-
* L. sulphureus *	Cui 12389	KR187106 ^a^	KX354487 ^a^	KX354519 ^a^	KX354561 ^a^	KX354608 ^a^	KX354653 ^a^
* L. sulphureus *	Cui 12388	KR187105 ^a^	KX354486 ^a^	KX354518 ^a^	KX354560 ^a^	KX354607 ^a^	KX354652 ^a^
* L. sulphureus *	Dai 12154	KF951295 ^a^	KF951302 ^a^	KX354521 ^a^	KX354563 ^a^	KX354610 ^a^	KX354655 ^a^
* L. sulphureus *	Z.R.L. CA04	KX354479 ^a^	KX354506 ^a^	KX354545 ^a^	KX354587 ^a^	KX354633 ^a^	KX354674 ^a^
* L. sulphureus *	Z.R.L. CA08	KX354480 ^a^	KX354507 ^a^	KX354546 ^a^	KX354588 ^a^	KX354634 ^a^	KX354675 ^a^
* L. sulphureus *	DA 41	EU40256	EU402533	-	EU402481	AB472660	-
* L. sulphureus *	TJV 99/150	EU402567	EU402530	-	EU402492	-	-
* L. sulphureus *	MAS 2	EU402568	EU402531	-	EU402491	-	-
* L. sulphureus *	JV 1106/15	KF951296 ^a^	KF951303 ^a^	KX354520 ^a^	KX354562 ^a^	KX354609 ^a^	KX354654 ^a^
* L. versisporus *	Cui 7882	KF951269 ^a^	KF951323 ^a^	-	KX354596 ^a^	KX354642 ^a^	KT894783 ^a^
* L. versisporus *	Li 15071314	KX354476 ^a^	KX357139 ^a^	-	KX354598 ^a^	KX354644 ^a^	KX354680 ^a^
* L. versisporus *	Dai 13160	KF951266 ^a^	KF951320 ^a^	-	KX354597 ^a^	KX354643 ^a^	KT894785 ^a^
* L. versisporus *	Yuan 6319	KX354475 ^a^	KX354503 ^a^	KX354541 ^a^	KX354583 ^a^	KX354629 ^a^	KX354670 ^a^
* L. versisporus *	Dai 10992	KF951272 ^a^	KF951325 ^a^	-	KX354600 ^a^	KX354646 ^a^	KX354681 ^a^
* L. versisporus *	Dai 13052	KF951271 ^a^	KF951324 ^a^	-	KX354601 ^a^	KX354647 ^a^	KX354682 ^a^
* L. xinjiangensis *	Dai 15825 (Paratype)	KX354465 ^a^	KX354493 ^a^	KX354527 ^a^	KX354569 ^a^	KX354616 ^a^	KX354661 ^a^
* L. xinjiangensis *	Dai 15828 (Paratype)	KX354461 ^a^	KX354489 ^a^	KX354523 ^a^	KX354565 ^a^	KX354612 ^a^	KX354657 ^a^
* L. xinjiangensis *	Dai 15953 (Holotype)	KX354460 ^a^	KX354488 ^a^	KX354522 ^a^	KX354564 ^a^	KX354611 ^a^	KX354656 ^a^
* L. xinjiangensis *	Dai 15898A (Paratype)	KX354464 ^a^	KX354492 ^a^	KX354526 ^a^	KX354568 ^a^	KX354615 ^a^	KX354660 ^a^
* L. zonatus *	HKAS 71806 (Paratype)	KF951284 ^a^	KF951310 ^a^	KX354548 ^a^	KX354590 ^a^	KX354636 ^a^	KT894796 ^a^
* L. zonatus *	Cui 10403 (Paratype)	KF951282 ^a^	KF951307 ^a^	KX354550 ^a^	KX354592 ^a^	KX354638 ^a^	-
* L. zonatus *	Cui 10404 (Holotype)	KF951283 ^a^	KF951308 ^a^	KX354551 ^a^	KX354593 ^a^	KX354639 ^a^	KT894797 ^a^

^a^ New sequences for this study.

Bayesian Inference (BI), Maximum Likelihood (ML) and Maximum Parsimony (MP) analyses were applied to the combined dataset. The best fit model of nucleotide evolution to each individual genetic marker and the combined dataset was selected with AIC (Akaike Information Criterion) using MrModeltest 2.3 (Posada and Crandall 1998, Nylander 2004). The best fit models were GTR for ITS, nrLSU, nrSSU, EF-1α, mtSSU, RPB2 and GTR+I+G for the combined dataset. The partitioned mixed model, which allows for model parameters estimated separately for each genetic marker, was used in the Bayesian analysis. BI was performed using MrBayes 3.1.2 (Ronquist and Huelsenbeck 2003) with 2 independent runs, each one beginning from random trees with 4 simultaneous independent chains, performing 4,000,000 replicates, sampling one tree every 100 generations. The first 25% of the sampled trees were discarded as burn-in and the remaining ones were used to reconstruct a majority rule consensus and calculate Bayesian posterior probabilities (BPP) of the clades.

ML searches were conducted with RAxML-HPC2 on Abe through the Cipres Science Gateway (www.phylo.org) and comprised 100 ML searches under the GTRGAMMA model, with all model parameters estimated by the programme. Only the maximum likelihood best tree from all searches was kept. In addition, 100 rapid bootstrap replicates were run with the GTRCAT model to assess the reliability of the nodes.

MP analysis was applied to the combined dataset as in Song and Cui (2017). Tree construction was performed in PAUP* version 4.0b10 (Swofford 2002) with the following settings. All characters were equally weighted and gaps were treated as missing data. Trees were inferred using the heuristic search option with TBR branch swapping and 1000 random sequence additions. Max-trees were set to 5000, branches of zero length were collapsed and all most parsimonious trees were saved. Clade robustness was assessed using a bootstrap analysis with 1000 replicates (Felsenstein 1985). The descriptive statistics of tree length (TL), consistency index (CI), retention index (RI), rescaled consistency index (RC) and homoplasy index (HI) were calculated for each most parsimonious tree generated.

Branches that received bootstrap support for maximum parsimony (MP), maximum likelihood (ML) and Bayesian posterior probabilities (BPP) greater than or equal to 75% (MP/ML) and 0.95 (BPP) were considered as significantly supported.

## Results

### Phylogenetic analyses

**Figure 1.**
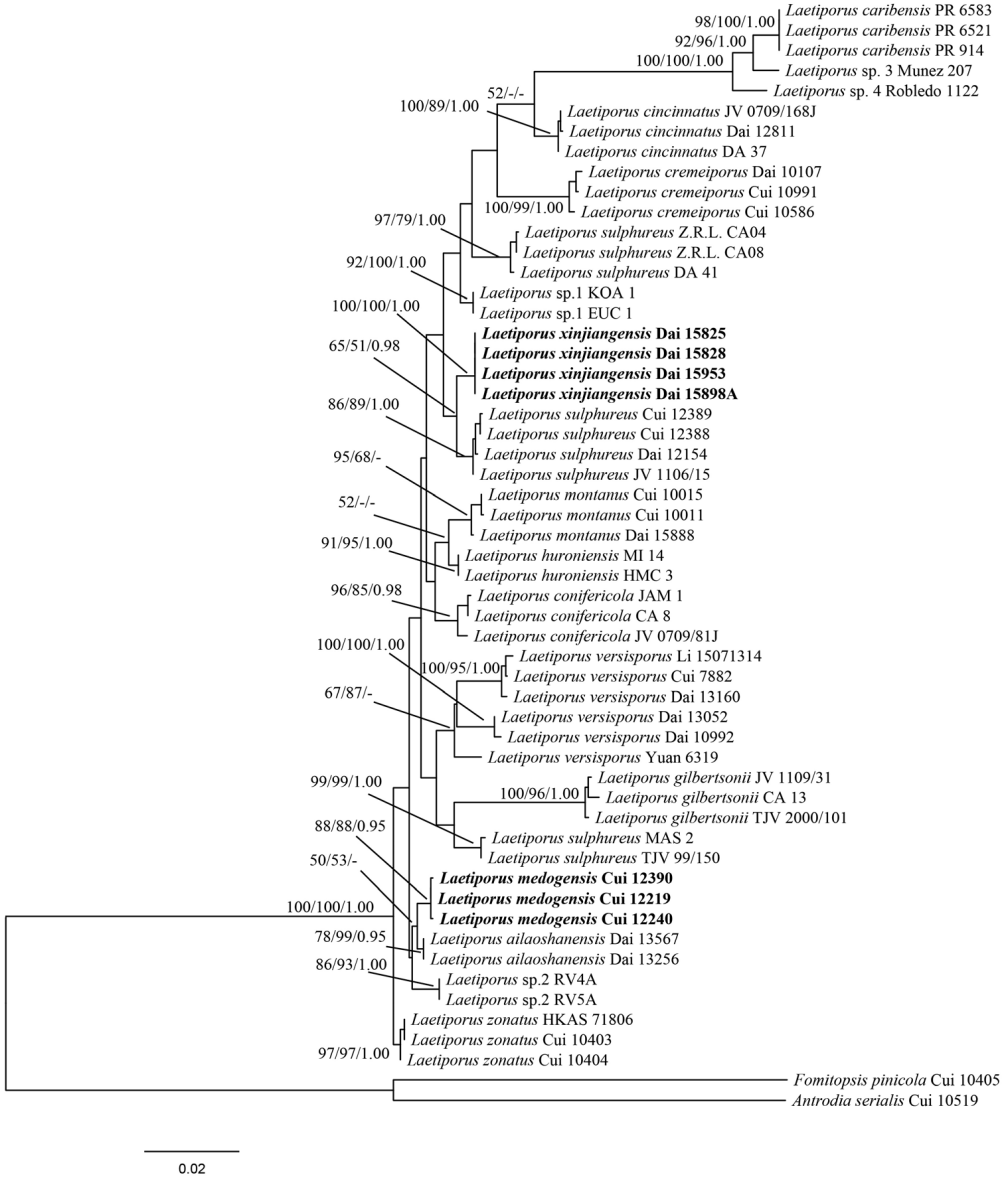
Strict consensus tree illustrating the phylogeny of *Laetiporus* generated by ML analysis based on ITS+nrLSU+nrSSU+mtSSU+EF-1α+RPB2 sequences. Branch support is indicated where MP/BS support is greater than 50% and collapsed below that support threshold. BPP is indicated when greater than 0.95. New species are indicated in bold.

The combined dataset (ITS+nrLSU+nrSSU+mtSSU+EF-1α+RPB2) included sequences from 55 samples representing 19 taxa. *Antrodiaserialis* and *Fomitopsispinicola* were used as outgroups. The dataset had a total aligned length of 3963 characters, of which 3137 (79.2%) were constant, 301 (7.6%) were variable and parsimony uninformative and 525 (13.2%) were parsimony informative. The parsimony analysis yielded 68 equally parsimonious trees (TL = 1173, CI = 0.812, RI = 0.865, RC = 0.702, HI = 0.188). The multiple sequence alignment and tree files were deposited at TreeBase (submission ID 21249; www.treebase.org). MP analysis and BI resulted in similar topologies as the ML analysis. The consensus tree inferred from the ML analysis with MP, ML and BPP values is shown in Figure 1.

Samples of *Laetiporus* clustered together with significant support (100% MP, 100% ML and 1.00 BPP; Figure 1). Sampled specimens of the two new species *L.medogensis* and *L.xinjiangensis* formed well-supported lineages (Figure 1).

### Taxonomy

#### Laetiporus medogensis

Taxon classificationFungiPolyporalesFomitopsidaceae

J. Song & B.K. Cui

MB821867

Figures 2a , 3

##### Diagnosis.

Differs from other *Laetiporus* species by its pinkish-buff to clay-buff pileal surface, buff-yellow pore surface and ellipsoid to ovoid basidiospores (5–6.2 × 4.2–5.2 μm).

##### Etymology.

*Medogensis* (Lat.): referring to the locality (Medog County) of the type specimens.

##### Holotype.

CHINA. Xizang Auto. Reg. (Tibet), Medog County, on living tree of *Abies*, 21 Sep 2014, Cui 12240 (BJFC 017154).

##### Basidiocarps.

Annual, sessile to laterally substipitate, imbricate, fleshy when fresh, crumbly when dry, without odour or taste. Pileus flabelliform to dimidiate, applanate, projecting up to 9 cm, 12 cm wide and 1 cm thick. Pileal surface pinkish-buff to clay-buff when fresh, becoming pale yellow upon drying, glabrous, azonate to faintly zonate. Margin soft and slightly viscous, fawn when juvenile, fading to reddish-brown when dry. Pore surface buff-yellow when fresh, becoming pale yellow to cream when dry; sterile margin cream when fresh, up to 3 mm wide; pores angular, 2–4 per mm; dissepiments thin, entire to lacerate. Context white when fresh, becoming cream to pale yellow when dry, up to 8.5 mm thick. Tubes concolorous with pore surface, crumbly or chalky, up to 1.5 mm long.

**Figure 2.**
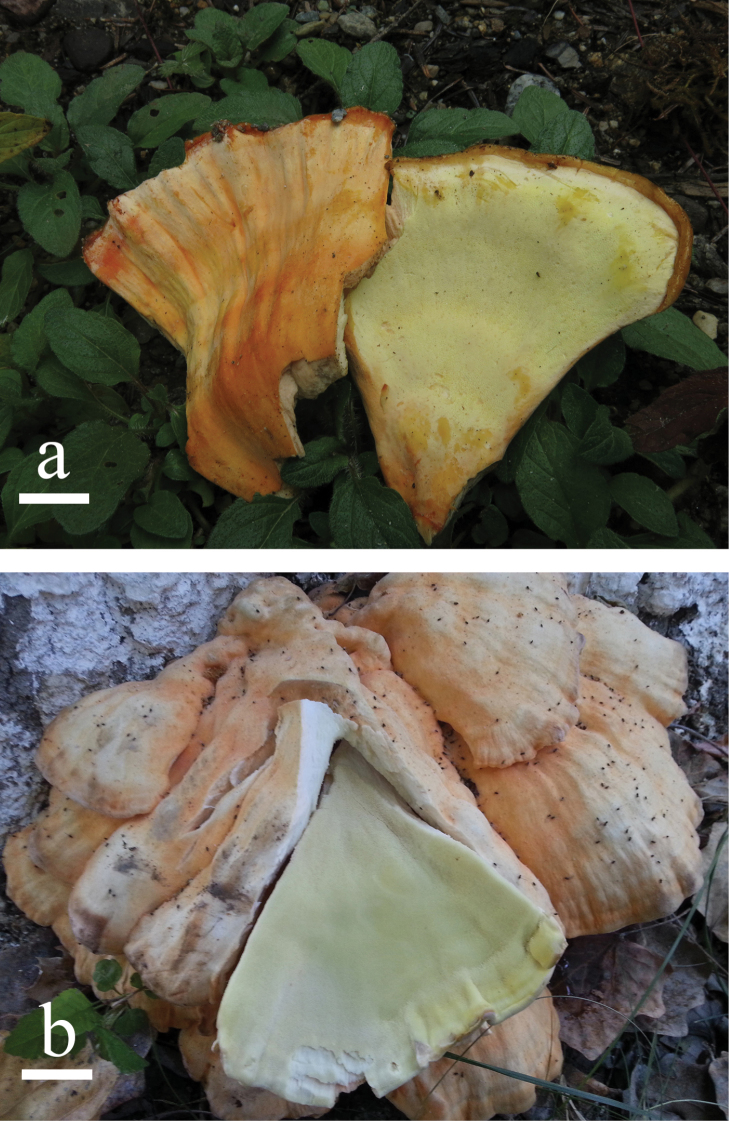
Basidiomata of *Laetiporus* species. **a***L.medogensis***b***L.xinjiangensis*. Scale bars: **a** = 2 cm, **b** = 3 cm.

##### Hyphal structure.

Hyphal system dimitic; generative hyphae simple-septate; skeletal hyphae IKI–, CB–, dissolving in KOH. Generative hyphae in context infrequent, hyaline, thin-walled, occasionally branched, up to 11 µm in diam.; skeletal hyphae in context dominant, thick-walled with a wide lumen, frequently branched and interwoven, occasionally simple-septate, hyaline, 4–11 μm in diam. Generative hyphae in tubes dominant, hyaline, thin-walled, frequently branched, simple-septate, 4–5 µm in diam.; skeletal hyphae in tubes thick-walled with a wide lumen, occasionally branched and simple-septate, subparallel along the tubes, 3–5 µm in diam.

##### Cystidia.

Cystidia and other sterile hyphal elements absent.

##### Basidia.

Basidia clavate, 20–25 × 8–9 μm, bearing four sterigmata and a basal simple-septum; basidioles clavate, smaller than basidia.

##### Spores.

Basidiospores ellipsoid to ovoid, hyaline, thin-walled, smooth, IKI–, CB–, 5–6.2 × 4.2–5.2 μm, L = 5.78 μm, W = 4.73 μm, Q = 1.22–1.23 (n = 60/2).

##### Additional specimens

**(paratypes) examined.** CHINA. Xizang Auto. Reg. (Tibet), Medog County, on living tree of *Abies*, 20 Sep 2014, Cui 12218 (BJFC 017132) & Cui 12219 (BJFC 017133); 21 Sep 2014, Cui 12241 (BJFC 017155); 24 Aug 2014, Cui 12390 (BJFC 017304).

**Figure 3.**
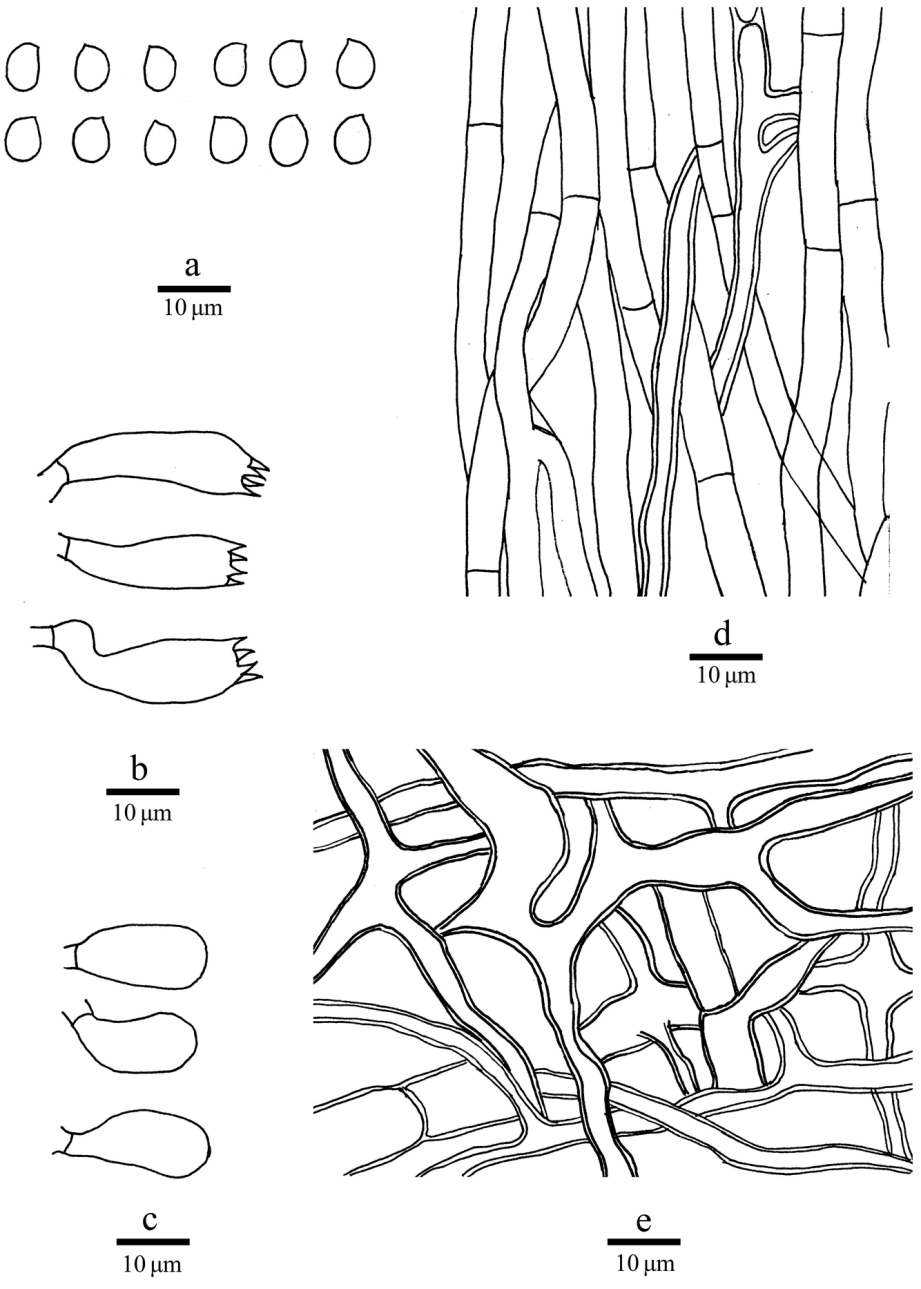
Microscopic structures of *Laetiporusmedogensis* (drawn from the holotype). **a** Basidiospores **b** Basidia **c** Basidioles **d** Hyphae from trama **e** Hyphae from context.

#### Laetiporus xinjiangensis

Taxon classificationFungiPolyporalesFomitopsidaceae

J. Song, Y.C. Dai & B.K. Cui

MB821868

Figures 2b , 4

##### Diagnosis.

Differs from other *Laetiporus* species by its pale-buff to clay-pink pileal surface, cream to light-yellow pore surface, large pores (2–3 per mm) and smaller basidiospores (4.5–5 × 3–4.2 μm).

##### Etymology.

*Xinjiangensis* (Lat.): referring to the locality (Xinjiang Autonomous Region) of the type specimens.

##### Holotype.

CHINA. Xinjiang Auto. Reg., Ili Kazak Autonomous Prefecture, Gongliu County, West Tianshan National Nature Reserve, on living tree of *Betula*, 14 Sep 2015, Dai 15953 (BJFC 020054).

##### Basidiocarps.

Annual, sessile to laterally substipitate, imbricate, odour distinctive, taste with acid flavor, fleshy when fresh, crumbly when dry. Pilei flabelliform to dimidiate, applanate, projecting up to 15 cm, 20 cm wide and 3 cm thick. Pileal surface pale-buff to clay-pink when fresh, becoming pale-buff to cream upon drying, glabrous, azonate to faintly zonate when fresh. Margin blunt, clay-buff to greyish-brown to brown when juvenile, fading to dark brown when dry. Pore surface cream to light yellow when fresh, becoming pale yellow when dry; sterile margin pale yellow when fresh, up to 2 mm wide; pores angular, 2–3 per mm; dissepiments thin, entire to lacerate. Context white when fresh, becoming cream to pale yellow when dry, up to 2.2 cm thick. Tubes concolorous with pore surface, crumbly or chalky, up to 8 mm long.

##### Hyphal structure.

Hyphal system dimitic; generative hyphae simple-septate; skeletal hyphae IKI–, CB–, dissolving in KOH. Generative hyphae in context infrequent, hyaline, thin-walled, occasionally branched, up to 11 µm in diam.; skeletal hyphae in context dominant, hyaline, thick-walled with a wide lumen, frequently branched and interwoven, occasionally simple-septate, 8–15 μm in diam. Generative hyphae in tubes dominant, hyaline, thin-walled, frequently branched, simple-septate, 4–6 µm in diam.; skeletal hyphae in tubes thick-walled with a wide lumen, occasionally branched and simple-septate, subparallel along the tubes or interwoven, 3–5 µm in diam.

##### Cystidia.

Cystidia and other sterile hyphal elements absent.

##### Basidia.

Basidia clavate, 20–25 × 6–8 μm, bearing four sterigmata and a basal simple-septum; basidioles clavate, smaller than basidia.

**Spores.** Basidiospores ellipsoid to ovoid, hyaline, thin-walled, smooth, IKI–, CB–, 4.5–5 × 3–4.2 μm, L = 4.87 μm, W = 3.65 μm, Q = 1.33–1.37 (n = 60/2).

##### Additional specimens

**(paratypes) examined.** CHINA. Xinjiang Auto. Reg., Shihezi, on living tree of *Populus*, 9 Sep 2015, Dai 15825 (BJFC 019930) & Dai 15828 (BJFC 019931); Burqin County, on living tree of *Salix*, 9 Sep 2015, Dai 15836 (BJFC 019937) & Dai 15838 (BJFC 019939); Burqin County, Kanas Integrated Nature Landscape Protect Region, on living tree of *Salix*, 11 Sep 2015, Dai 15893 (BJFC019994); Ili Kazak Autonomous Prefecture, on living tree of *Populus*, 13 Sep 2015, Dai 15902 (BJFC 020003) & Dai 15905 (BJFC 020006); 4 Oct 2015, Dai 15898A (BJFC 019999).

**Figure 4.**
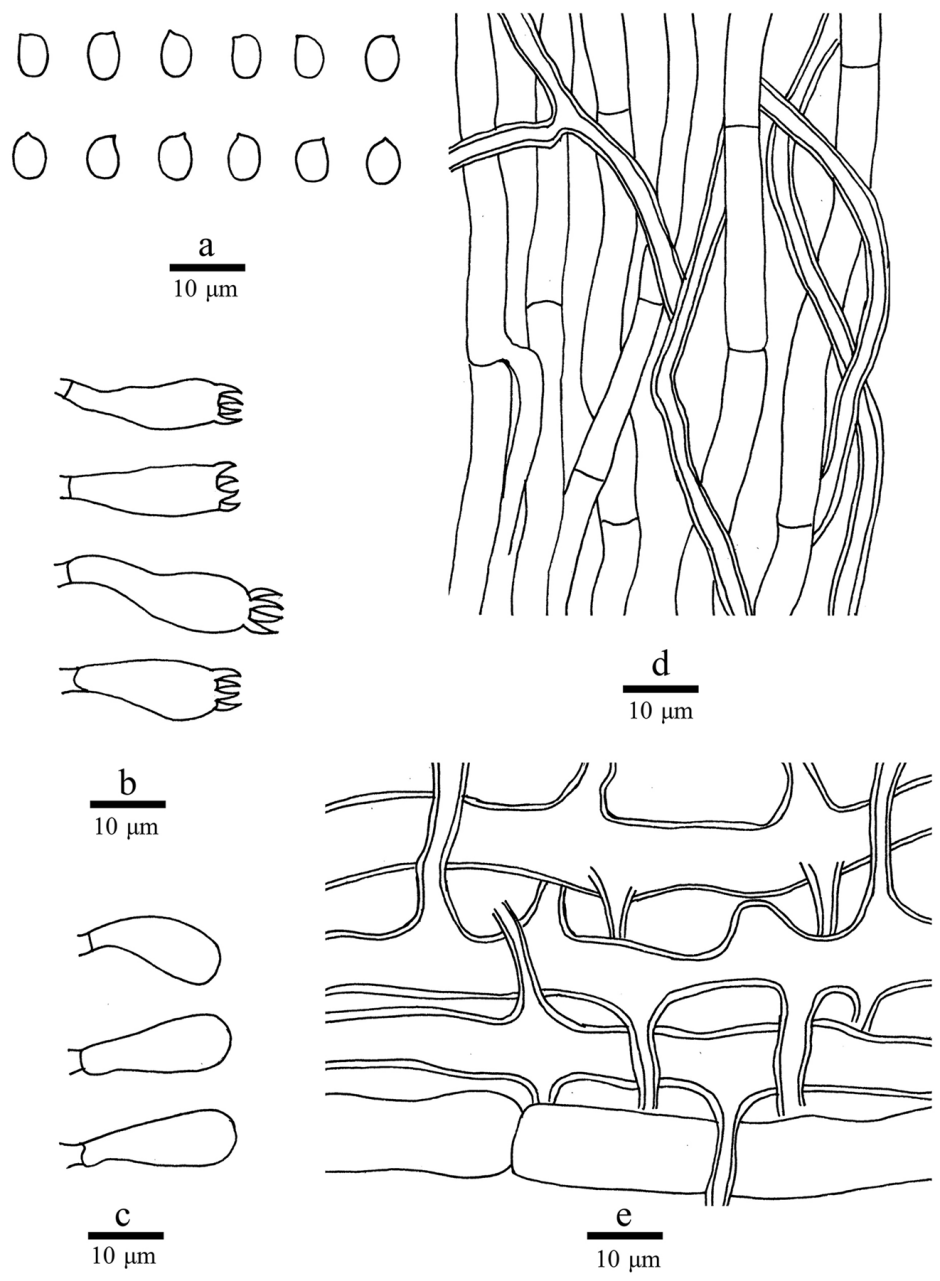
Microscopic structures of *Laetiporusxinjiangensis* (drawn from the holotype). **a** Basidiospores **b** Basidia **c** Basidioles **d** Hyphae from trama **e** Hyphae from context.

## Discussion

Recent studies indicated that *Laetiporussulphureus* in East Asia is a species complex, comprising several morphologically and ecologically distinct species (Ota et al. 2009, Song et al. 2014). The current study recognised two new species, namely, *L.medogensis* and *L.xinjiangensis* and, altogether, eight *Laetiporus* species have been found in China thus far. The multi-gene phylogenetic topology showed that the new species formed two separate lineages (Figure 1).

*Laetiporusmedogensis* and *L.ailaoshanensis* group together with moderate to low MP and ML support (50% MP and 53% ML). Both *L.medogensis* and *L.ailaoshanensis* are found in Southwest China. Morphologically, *L.ailaoshanensis* is similar to *L.medogensis* by producing orange to yellow pileal surface, white context and ellipsoid to ovoid basidiospores. However, *L.medogensis* is found on conifers and the pore surface is yellow; *L.ailaoshanensis* grows on hardwoods and has a white pore surface (Song et al. 2014). *L.sulphureus* resembles *L.medogensis* by producing yellow to orange pileal surface and yellow pore surface; however, *L.sulphureus* usually grows on hardwoods and produces thicker basidiocarps and larger basidiospores (5–7 × 4–5 µm; Ota et al. 2009). *L.versisporus* and *L.medogensis* share similar characters including yellow pileal surface, yellowish pore surface and ovoid to ellipsoid basidiospores; however, *L.versisporus* differs from *L.medogensis* in having smaller pores (2–6 per mm) and larger basidiospores (4–6.8 × 3–5.5 μm). In addition, *L.versisporus* grows on hardwoods and is mainly distributed in subtropical to tropical areas (Ota et al. 2009, Song and Cui 2017).

*Laetiporusxinjiangensis*, *L.sulphureus* and *L.montanus* are all common in Northwest China. Both *L.xinjiangensis* and *L.sulphureus* grow on angiosperms and group together in the phylogenetic tree with moderate MP, ML and significant BI support (65% MP, 51% ML and 0.98 BPP). Morphologically, *L.sulphureus* is similar to *L.xinjiangensis* in having yellowish pore surface and ovoid to ellipsoid basidiospores; however, *L.sulphureus* produces larger basidiospores (5–7 × 4–5 μm) and has smaller pores (2–5 per mm; Burdsall and Banik 2001). *L.montanus* is similar to *L.xinjiangensis* by producing a burlywood pileal surface and a yellowish pore surface; however, *L.montanus* differs by producing pyriform basidiospores (6–8 × 4–5.5 μm) and by growing on gymnosperms (Tomšovský and Jankovský 2008).

Our research expanded the number of *Laetiporus* species to 17 around the world. However, studies in the Southern Hemisphere are still few and the relationships amongst *Laetiporus* species remain unresolved (Lindner and Banik 2008, Pires et al. 2016, Song and Cui 2017). More comprehensive studies on *Laetiporus* depend on more collections and data from poorly sampled areas. The main morphological characters, host trees and distribution areas of species in the *L.sulphureus* complex are provided in Table 2. An identification key to the known species of *Laetiporus* is provided.

### Key to accepted species in the *Laetiporussulphureus* complex

**Table:** 

1	Pore surface light goldenrod to sulphur yellow or light yellow when fresh	**2**
–	Pore surface cream to white when fresh	**10**
2	Occurring on conifers	**3**
–	Occurring on hardwoods	**6**
3	Distributed in cool temperate to boreal zones in East Asia and Europe	**4**
–	Distributed in North America	**5**
4	Basidiospores 6–8 × 4–5.5 µm	*** L. montanus ***
–	Basidiospores 5–6.2 × 4.2–5.2 μm	*** L. medogensis ***
5	Basidiospores 5–7 × 3.8–5 µm; distributed in eastern North America	*** L. huroniensis ***
–	Basidiospores 6.5–8 × 4–5 µm; distributed in far western North America	*** L. conifericola ***
6	Pores 4–5 per mm	*** L. caribensis ***
–	Pores 2–4 per mm	**7**
7	Basidiocarps single, occasionally imbricate but not in large clusters; anamorphic form frequently produced	*** L. versisporus ***
–	Basidiocarps imbricate, rarely single; no anamorphic form or rarely produced	**8**
8	Basidiospores 4.5–5 × 3–4.2 µm	*** L. xinjiangensis ***
–	Basidiospores 5–7 × 3–5.5 µm	**9**
9	Distributed in temperate zones	*** L. sulphureus ***
–	Distributed in temperate to tropical zones	*** L. gilbertsonii ***
10	Basidiocarps arising from soil or surface of roots near the base of living trees	*** L. cincinnatus ***
–	Basidiocarp arising from trunks of standing trees or on fallen logs	**11**
11	Distributed in mountain forests of subtropical zones	*** L. ailaoshanensis ***
–	Distributed in cool temperate to boreal zones	**12**
12	Pileal surface cream to white, pores 3–6 per mm	*** L. zonatus ***
–	Pileal surface light orange to reddish-orange, pores 2–4 per mm	*** L. cremeiporus ***

**Table 2.** The main morphological characters, host trees and distribution areas of species in the *Laetiporussulphureus* complex.

Species	Pileal surface	Pore surface	Pores	Basidiospores	Distribution	Host	References
* L. ailaoshanensis *	orange yellow to reddish orange	cream to buff	3–5/mm	ovoid to ellipsoid 5.0–6.2 × 4.0–5.0 µm	subtropical areas of south-western China	* Lithocarpus *	Song et al. 2014
* L. caribensis *	orange to pale orange	lemon yellow	4–5/mm	ellipsoid 4.0–4.5 × 2.7–3.6 µm	tropical zones of the Caribbean basin and central America	*Guareaguidonia*, *Dacryodes*	Banik et al. 2012
* L. cincinnatus *	bright salmon orange	pale cream	2–4/mm	broadly ovoid 4.5–5.5 × 3.5–4.0 µm	throughout the eastern USA except for in the states along the Gulf of Mexico, common in the Great Lakes regions	arising from the soil (*Quercus*)	Burdsall and Banik 2001
* L. conifericola *	bright orange to salmon orange	lemon yellow to bright creamy yellow	2–4/mm	broadly ovoid 6.5–8.0 × 4.0–5.0 µm	western North America from California to Alaska	*Tsuga*, *Picea*, *Abies*, *Pinus*	Burdsall and Banik 2001
* L. cremeiporus *	light orange to reddish-orange	yellowish-white to cream	2–4/mm	ovoid to ellipsoid 5.6–7.0 × 3.9–4.7 µm	cool temperate to boreal areas of East Asia	*Quercus*, *Pyrus*, *Prunus*	Ota et al. 2009
* L. gilbertsonii *	pale salmon orange or pale pinkish-orange	lemon yellow to pale lemon yellow (in West USA) or isabelline to nearly white (in Southeast USA)	2–4/mm	broadly ovoid 5.0–6.5 × 3.5–4.5 µm	North America, Central and South America	*Eucalyptus*, *Quercus*, *Prunus*	Burdsall and Banik 2001, Banik et al. 2012
* L. huroniensis *	bright orange	lemon yellow	2–4/mm	broadly ovoid 5.0–7.0 × 4.2–5.0 µm	eastern North America and in its Great Lakes areas	* Tsuga *	Burdsall and Banik 2001
* L. medogensis *	pinkish-buff to clay-buff	buff-yellow	2–4/mm	ellipsoid to ovoid 5–6.2 × 4.2–5.2 μm	cool temperate areas of south-western China	* Abies *	in the present study
* L. montanus *	light orange to reddish-orange	bright sulphurous yellow	1–4/mm	pyriform or ovoid to ellipsoid 6.0–8.0 × 4.0–5.5 µm	boreal zones in north-eastern China and in mountain areas of Japan and Central Europe	*Picea*, *Larix*, *Abies*	Tomšovský and Jankovský 2008, Ota et al. 2009
* L. sulphureus *	bright salmon orange	lemon yellow	2–4/mm	ovoid to ellipsoid 5.0–6.8 × 4.0–5.0 µm	North America, Europe and South America	*Acer*, *Salix*, *Gleditisa*, *Quercus*, *Fraxinus*, *Castanea*, *Salix*	Burdsall and Banik 2001
* L. versisporus *	whitish to sulphur yellow	usually yellow, sometimes pale yellow to nearly white	3–6/mm	ovoid to short ellipsoid 4.0–6.8 × 3.0–5.5 µm	cool temperate to tropical areas of East Asia	*Robinia*, *Castanea*, *Quercus*, *Elaeocarpus*, *Castanopsis*	Núñez and Ryvarden 2001, Ota et al. 2009
* L. xinjiangensis *	pale-buff to clay-pink	cream to light yellow	2–3/mm	ellipsoid to ovoid 4.5–5 × 3–4.2 μm	temperate areas of western China	*Betula*, *Populus*, *Salix*	in the present study
* L. zonatus *	white to cream and buff to clay-buff at base	white to cream	2–5/mm	ellipsoid to pyriform or drop-shaped 5.8–7.2 × 4.3–5.5 µm	high mountains of temperate areas of south-western China	* Quercus *	Song et al. 2014

## Supplementary Material

XML Treatment for Laetiporus medogensis

XML Treatment for Laetiporus xinjiangensis
